# scTRIM44 Positively Regulated Siniperca Chuatsi Rhabdovirus Through RIG-I- and MDA5-Mediated Interferon Signaling

**DOI:** 10.3390/v16121876

**Published:** 2024-12-02

**Authors:** Yinjie Niu, Xinmei Yang, Hongru Liang, Xia Luo, Baofu Ma, Qiang Lin, Xiaozhe Fu, Ningqiu Li

**Affiliations:** Ministry of Agriculture and Rural Affairs, Guangdong Provincial Key Laboratory of Aquatic Animal Immunology and Sustainable Aquaculture, Key Laboratory of Fishery Drug Development, Pearl River Fisheries Research Institute, Chinese Academy of Fishery Sciences, Guangzhou 510380, China; niuyinjie0530@163.com (Y.N.); yxm122623@163.com (X.Y.); hrliang13@126.com (H.L.); lxwenhao@163.com (X.L.); mabf@prfri.ac.cn (B.M.); lin9902057@163.com (Q.L.); fuxiaozhe-1998@163.com (X.F.)

**Keywords:** scTRIM44, LMBV, ISKNV, SCRV, mandarin fish

## Abstract

Tripartite Motif-Containing 44 (TRIM44) is responsible for cancers, neurodegenerative diseases, and viral infections. However, the role of *Siniperca chuatsi* TRIM44 (scTRIM44) during viral infection remains unclear. In the present study, we analyzed the molecular characteristics of scTRIM44 and its role in infectious spleen and kidney necrosis virus (ISKNV), largemouth bass virus (LMBV), and Siniperca chuatsi rhabdovirus (SCRV) infection. ScTRIM44 contained one B-box domain (B, 166–207 aa) and a coiled-coil domain (CC, 279–309 aa), but lacked the canonical RING domain of E3 ubiquitin ligases. The scTRIM44 mRNA was expressed relatively high in immune-related tissues. The mRNA expression of scTRIM44 significantly decreased in vivo and vitro post-ISKNV and -LMBV infection. However, the expression of scTRIM44 mRNA showed significant up-regulation post-SCRV infection. ScTRIM44 positively regulated SCRV infection in CPB cells, but copies of ISKNV and LMBV showed no significant alteration in over-expressed or knocked-down scTRIM44 cells. Moreover, scTRIM44 positively regulated RIG-I- and MDA5-mediated interferon molecule signaling. These data suggested that scTRIM44 promoted SCRV infection by positively regulating RIG-I- and MDA5-mediated interferon molecule signaling, but didn’t regulate ISKNV and LMBV infection. This research provided a comprehensive insight into the antiviral activity of scTRIM44.

## 1. Introduction

The mandarin fish (*Siniperca chuatsi*) is a universally cultured species in Asian countries with delicious taste, abundant nutrition, and high market value [1]. Infectious spleen and kidney necrosis virus disease (ISKNVD), Siniperca chuatsi rhabdovirus disease (SCRVD), and largemouth bass virus disease (LMBVD) are major viral diseases; they have caused significant economic losses and hindered the sustainable development of the mandarin fish industry [2,3,4,5]. ISKNV and LMBV are both enveloped, icosahedral double-stranded DNA viruses, which belong to the *Iridoviridae* family [6,7]. SCRV is a negative-sense single-stranded RNA, which belongs to the *Rhabdoviridae* family [8]. The tripartite motif (TRIM) proteins induced by IFNs play an important role in the mammalian immune defense against microbial pathogens infection [9].

The TRIM protein family is characterized by three conserved N-terminal domains (named RBCC domains), including a canonical RING domain (R), one or two B-box domains (B), and one coiled-coil domain (CC) [10,11]. TRIM proteins containing the RING-finger domain are defined as the E3 ubiquitase. Recently, the expression of TRIM genes in many fish has been shown to become induced by viral infection or poly I:C in vitro and in vivo, including TRIM8, TRIM13, TRIM16L, TRIM32, TRIM35, TRIM39, TRIM62, and TRIM47 [12,13]. TRIM23 regulated RIG-I/MDA5-mediated antiviral innate response through ubiquitination. *Siniperca chuatsi* TRIM59 (scTRIM59) negatively regulated ISKNV, SCRV infection, and IRF3/IRF7-mediated signal genes [14]. The tripartite motif-containing 44 (TRIM44) protein is a new member of the TRIM family, which was cloned from a mouse brain cDNA library in 2001 [15]. TRIM44 has a zinc finger domain in the N-terminal region, but lacks the RING finger domain, it is an atypical TRIM family protein with the activity of ubiquitin-specific proteases (USps) [16]. Many studies reported that TRIM44 is involved in multiple disorders such as neurodegenerative diseases, cancers, and viral infections [17,18]. Yang et al. demonstrated that TRIM44 promoted virus-triggered IFN production through increasing the RIG-mediated signaling pathway [19]. When a viral pathogen entered the body, both RIG-1 and MDA5 caused the conformational change after recognizing the released RNA. Subsequently, the RIG-1 and MDA5 interacted with the Virus-Induced Signaling Adapter (VISA), which can activate IRF3 and IRF7 to promoting the production of type I interferons [20]. Zheng et al. reported that the expression of Singapore grouper iridovirus (SGIV) MCP and VP19 had no significant changes, but the CP and RdRp expression of red-spotted grouper nervous necrosis virus (RGNNV) was significantly up-regulated in EcTRIM44-over-expressing cells [21]. However, the molecular characteristics and antiviral activities of scTRIM44 are not fully understood.

In this study, we analyzed the molecular characteristics and structures of scTRIM44, and explored the changes in scTRIM44 mRNA post-ISKNV, -LMBV, and -SCRV infection. Moreover, we elucidated the antiviral activity of scTRIM44 in ISKNV, LMBV, and SCRV infection, and the regulation of scTRIM44 on interferon-mediated signaling molecules. These data provided a novel insight into the antiviral activity of scTRIM44 through interferon response signaling.

## 2. Materials and Methods

### 2.1. Cells and Virus Strains

We constructed the CPB (Chinese perch brain cell line) cells and cultured them in L-15 medium with 8% FBS [22]. The LMBV, ISKNV, and SCRV virus strains were isolated in our laboratory [23,24].

### 2.2. Molecular Analysis of scTRIM44

The nucleotide sequence of the scTRIM44 coding region was translated into an amino acid (aa) sequence with MEGA7.0.26. The structural characteristics of scTRIM44 amino acids were predicated by TMHMM (http://www.cbs.dtu.dk/services, accessed on 9 August 2023).

The aa sequences of scTRIM44 and 17 other fish species were aligned by using Clustal W. Then, we constructed the phylogenetic tree of 18 scTRIM44 protein sequences by using the neighbor-joining algorithm of MEGA 7.0.26 (bootstrap replications: 1000).

### 2.3. The Distribution of scTRIM44 mRNA in Mandarin Fish

To investigate the expression of scTRIM44 mRNA in healthy fish, the brain, heart, liver, stomach, hemocyte, intestine, head kidney, trunk kidney, and spleen were collected from three healthy fish (9 ± 0.5 cm). These organs were chosen based on immunological functions and the targets for replication by the three chosen viruses [24]. The scTRIM44 mRNA was detected with the SYBR Premix Ex Taq kit (Takara Bio, Ostsu, Japan).

### 2.4. The Changes in scTRIM44 mRNA Post-SCRV, -ISKNV, and -LMBV Infection

Ninety mandarin fish (9 ± 1 cm) were divided into three groups (A, B, C). First, three fish of each group were taken out as the negative sample at 0 h; then the remaining fish in the A, B, and C groups were injected with SCRV (10^4^ TCID50/fish), ISKNV (10^5^ TCID50/fish), or LMBV (10^4^ TCID50/fish), respectively. The spleens of fish infected with SCRV were collected at 0, 3, 6, 12, 24, 48, and 72 h post-infection. The spleens of fish infected with ISKNV were collected at 0, 3, 6, 12, 24, 48, 72, 96, and 168 h post-infection. The spleens of fish infected with LMBV were collected at 0, 3, 6, 12, 24, 48, 72, 96, and 120 h post-infection. Three fishes were dissected at each indicated time.

The CPB cells were incubated with SCRV (MO1 = 0.1), ISKNV (MO1 = 1), and LMBV (MOI = 0.1). Three parallel replicate cultures of CPB cells were collected at 0, 1, 2, 4, 6, and 8 h post-SCRV infection, respectively. Three parallel wells were collected at 0, 2, 4, 6, 8, 12, 48, and 72 h post-ISKNV infection, respectively. The CPB cells infected with LMBV were gathered at 0, 3, 6, 12, 24, 48, and 72 h post-infection.

### 2.5. The Effect of scTRIM44 on SCRV, ISKNV, and LMBV Infection

The siRNAs were synthesized at Genepharma. scTRIM44 was knocked down by transfecting 25 nM siRNA with TransIntro^TM^ EL transfection reagent according to the manufacturer’s instructions. The RNA of cells transfected with siRNAs were extracted and reversed, and gene targets amplified and quantified by qPCR. Then, the cells transfected with siRNA were infected with ISKNV (MOI = 0.1), SCRV (MOI = 0.001), and LMBV (MOI = 0.01), respectively.

The CDs (coding sequences, CDs) of scTRIM44 were amplified by PCR, then ligated to the parallel eukaryotic expression vector pCMV-EGFR-scTRIM44. The over-expressing scTRIM44 cells were constructed by transfecting pCMV-EGFP-scTRIM44 with FuGENE^®^6 and selecting with 0.1 mg/mL geneticin. Then, over-expressed scTRIM44 cells were infected with ISKNV (MOI = 0.1), SCRV (MOI = 0.001), and LMBV (MOI = 0.01), respectively.

### 2.6. TaqMan Real-Time PCR and Quantitative RT-PCR

We detected the copies of SCRV RNA, ISKNV DNA, and LMBV DNA with TaqMan PCR methods. TaqMan-PCR amplification was performed as follows: 2 × Premix (Takara, Japan) Ex Taq™ 10 μL, forward primer 0.4 μL, and reverse primer 0.4 μL, probe 0.4 μL, 50 × Rox Reference Dye II 0.4 μL, DNA 2 μL, and ddH_2_O 6.4 μL.

The relative expression level of scTRIM44 mRNA was detected by quantitative RT-PCR, then was calculated by using a 2^−ΔΔCT^ method relative to mean expression in uninfected fish or uninfected CPB cells (time point 0); the 18S rRNA was used as the reference gene. The used primers are listed as Table 1.

### 2.7. Statistical Analysis

The fish and cell experiments were performed in triplicate. Statistical data were analyzed by one-way analysis of variance (ANOVA) (expressed as mean ± SD). All data were compared using SPSS 13.0 (SPSS, Chicago, IL, USA). The relative expression of scTRIM44 expression was relative to time-zero. *p* < 0.05 represented a statistical difference, *p* < 0.01 represented a significant difference, *p* < 0.001 represented an extremely significant difference.

## 3. Results

### 3.1. Molecular Organization and Characteristics of ScTRIM44

**Figure 1:**
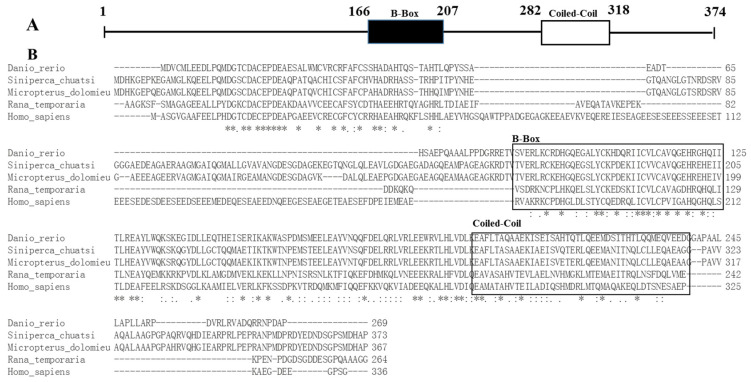
The molecular characteristics of scTRIM44. A: The structure diagram of scTRIM44. B: The aa sequence and structure component of scTRIM44. * represented the identity; . and : represented the similarity.

**Figure 2:**
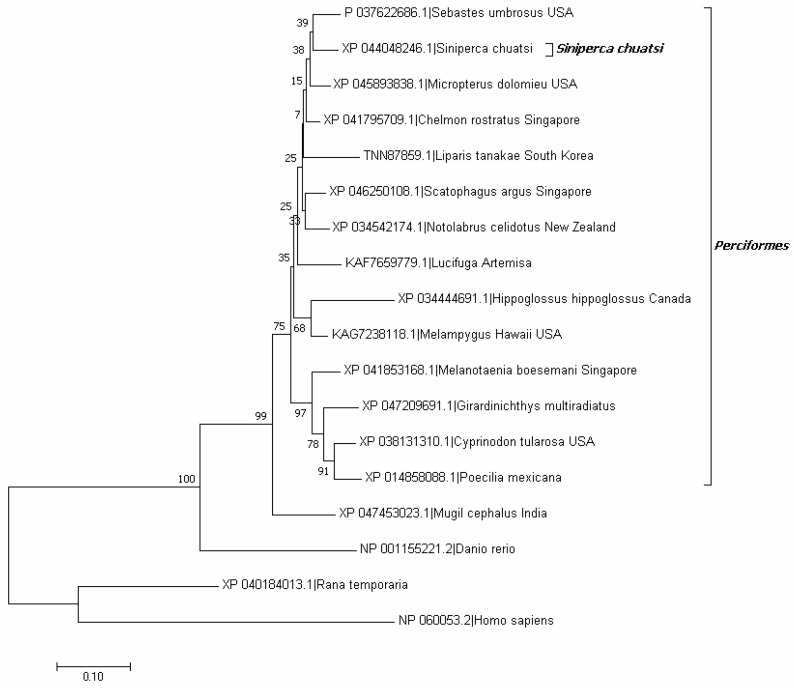
The phylogenetic tree of 18 TRIM44 aa sequences constructed by using MEGA 7.0.

Multiple sequence alignment indicated that TRIM44 differed considerably among different species (Figure 1). Phylogenetic analysis suggested that the relative of scTRIM44 was close to *Sebastes umbrosus* and *Micropterus dolomieu* and belonged to the *Perciformes* branch (Figure 2). The TRIM44 identity in the Perciformes branch was 44.23%, and the conservative regions were predominately located in the C-terminal (1–83 aa) and 157–328 aa. The identity was 90.90%, 42.89%, 26%, and 23% between scTRIM44 and TRIM44 of *Micropterus dolomieu*, *Danio rerio*, *Rana temporaria*, and *Homo sapiens*, respectively (Figure 1).

The trans-membrane analysis indicated that scTRIM44 was an intracellular protein. It contained a B-box-type zinc finger superfamily (Bbox_SF), one B-box domain (B, 166–207 aa), and a coiled-coil domain (CC, 279–309 aa), but lacked the canonical RING domain. The domains (B and CC) of scTRIM44 were 95.77%, 70.43, 56.34%, and 33.81% identical with *Micropterus dolomieu*, *Danio rerio*, *Rana temporaria* and *Homo sapiens*, respectively (Figure 1). The structure of scTRIM44 was similar to mammalian TRIM44, which indicated that scTRIM44 had similar antiviral function of mammalian TRIM44.

### 3.2. Expression of scTRIM44 in Siniperca chuatsi

**Figure 3:**
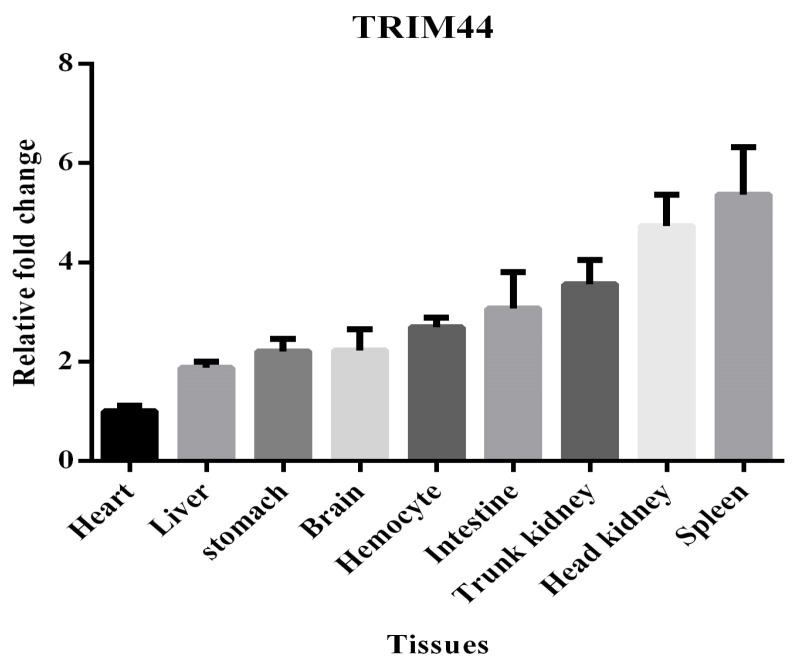
The scTRIM44 mRNA expression in different tissues of mandarin fish. The relative fold change of scTRIM44 was calculated relative to that of the heart.

The expression of scTRIM44 mRNA can be detected in the heart, liver, stomach, brain, hemocyte, intestine, trunk kidney, head kidney, and spleen. Especially, the expression of scTRIM44 mRNA was relatively abundant in immune organs, including the head kidney, trunk kidney, and spleen. This result indicated that scTRIM44 plays an important role in the immune function of Mandarin fish (Figure 3).

### 3.3. The Effect of SCRV Infection on scTRIM44

**Figure 4:**
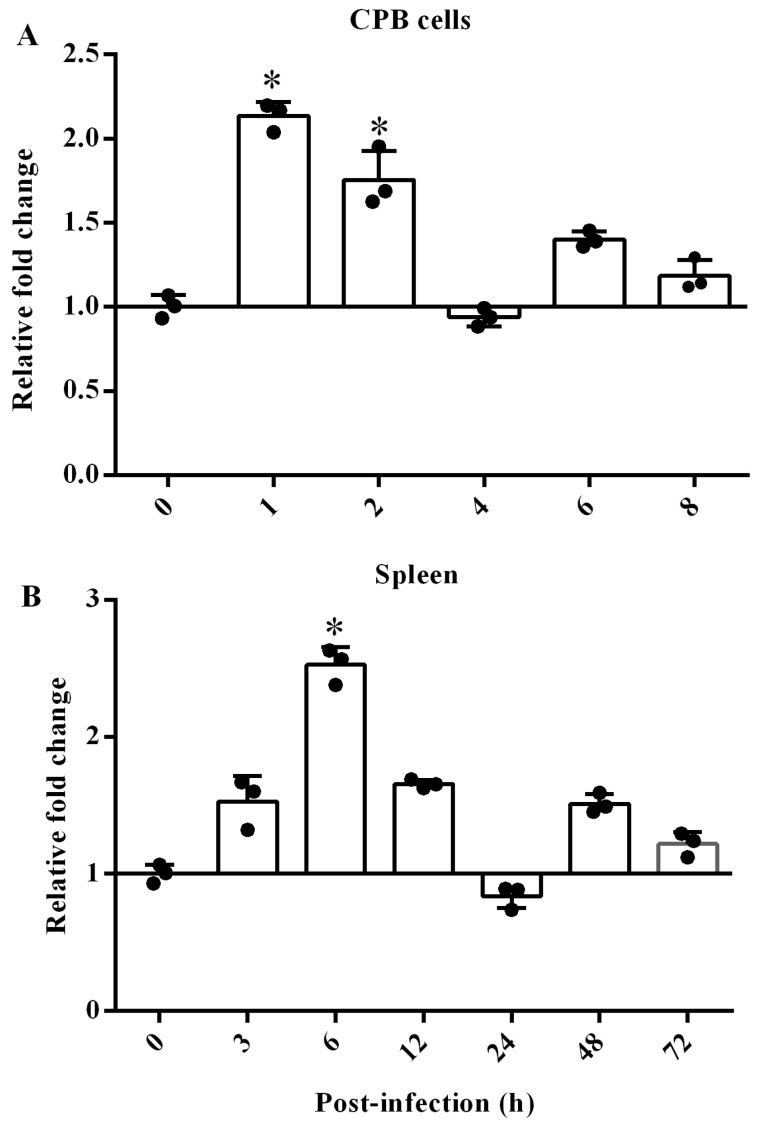
The expression of scTRIM44 significantly increased post-SCRV infection. (**A**): scTRIM44 in cells infected with SCRV was detected by using qRT-PCR; the expression of scTRIM44 represented up-regulation at 1, 2, 6, and 8 h in CPB cells post-SCRV infection. (**B**): The scTRIM44 mRNA also was up-regulated at 3, 6, 12, 48, and 72 h in spleen post-SCRV infection. *: *p* < 0.05.

The changes of scTRIM44 mRNA in spleen and CPB cells were detected by qRT-PCR. The expression of scTRIM44 mRNA was rapidly up-regulated at 1 and 2 h, and it increased slightly at 6 and 8 h in CPB cells post-SCRV infection (Figure 4A); The scTRIM44 mRNA significantly increased at 6 h, and it showed a slight up-regulation at 3, 12, 48, and 72 h in spleen post-SCRV infection (Figure 4B). Those results indicated that the expression was up-regulated in vivo and vitro post-SCRV infection.

### 3.4. The Changes in scTRIM44 Post-ISKNV or -LMBV Infection

**Figure 5:**
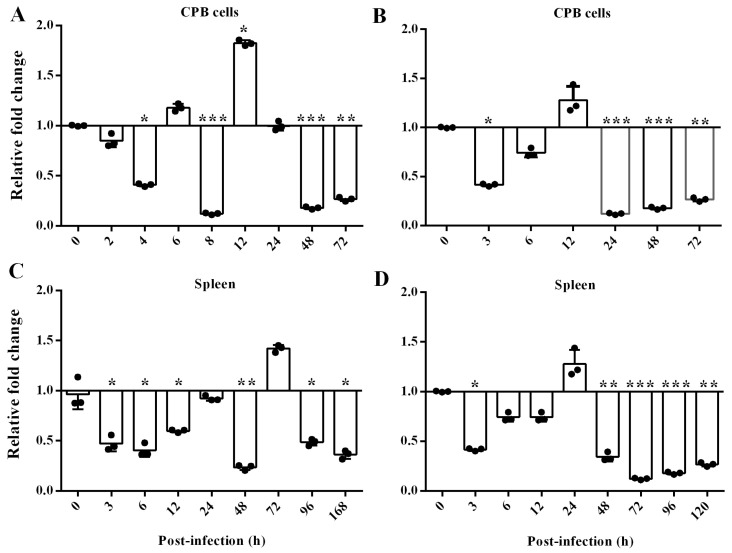
The response of scTRIM44 mRNA was significantly down-regulated post-ISKNV and LMBV infection. (**A**): The expression of scTRIM44 mRNA was significantly down-regulated at 4, 8, 48, and 72 h in CPB cells; (**C**): The scTRIM44 mRNA significantly decreased at 3, 6, 12, 48, 96, and 168 h in spleen after ISKNV infection; (**B**): The expression of scTRIM44 mRNA markedly decreased in CPB cells at 3, 24, 48, and 72 h post-LMBV infection; (**D**): The scTRIM44 mRNA significantly decreased in spleen at 3, 48, 72, 96, and 120 h post-LMBV infection. The changes in scTRIM44 were detected by using qRT-PCR post-ISKNV infection, *: *p* < 0.05; **: *p* < 0.01; ***: *p* < 0.001.

To determine the changes of scTRIM44 mRNA induced by ISKNV or LMBV infection, we analyzed the expression of scTRIM44 mRNA in CPB cells and spleen post-ISKNV or LMBV infection, respectively. In CPB cells, the expression of scTRIM44 mRNA was significantly reduced at 4, 8, 48, and 72 h and the expression of scTRIM44 mRNA showed a slightly down-regulation at 6 h, but scTRIM44 mRNA was significantly up-regulated at 12 h post-ISKNV infection (Figure 5A). In spleen, the expression of scTRIM44 mRNA was markedly down-regulated at 3, 6, 12, 48, 96, and 168 h, and the scTRIM44 mRNA slightly increased at 72 h post-ISKNV infection (Figure 5B). Overall, the expression of scTRIM44 mRNA was significantly down-regulated in vivo and in vitro post-ISKNV infection.

In cells, the expression of scTRIM44 mRNA was significantly decreased at 3, 24, 48, and 72 h, and the scTRIM44 mRNA was slightly down-regulated at 6 h post-LMBV infection. In spleen, the expression of scTRIM44 mRNA was markedly down-regulated at 3, 48, 72, 96, and 120 h, and the expression of scTRIM44 mRNA showed a slight down-regulation at 6 and 12 h post-LMBV infection. This result indicated that expression of scTRIM44 mRNA presented a significantly down-regulation in cells and spleen post-LMBV infection.

### 3.5. scTRIM44 Positively Regulated SCRV Infection but Did Not Regulate ISKNV and LMBV Infection

**Figure 6:**
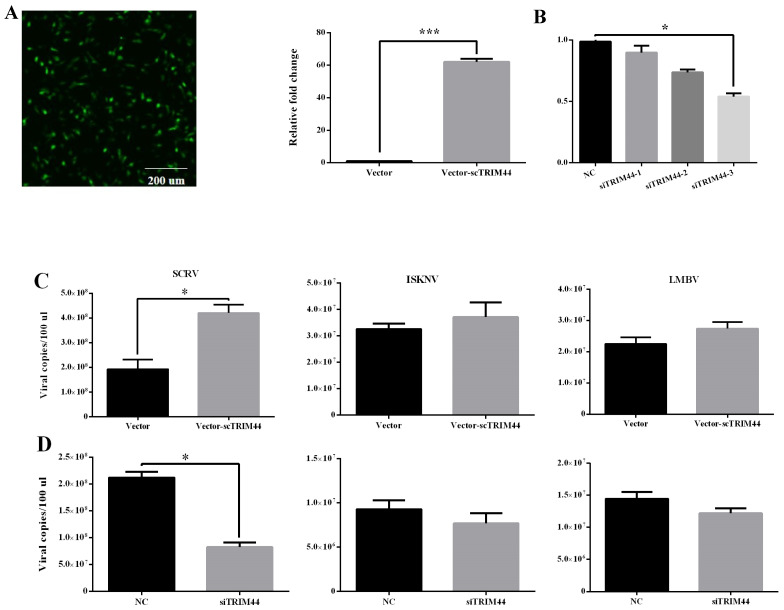
scTRIM44 positively regulated SCRV infection but didn’t regulate ISKNV and LMBV infection. (**A**): The green fluorescence showed that in over-expressing TRIM44 cells, the scTRIM44 mRNA in over-expressing scTRIM44 cells was significantly up-regulated compared to the vector group. (**B**): The expression of TRIM44 mRNA was markedly down-regulated in cells transfected with siTRIM44-3. (**C**): The viral copies of the SCRV genome significantly decreased in over-expressing scTRIM44 cells, and the viral copies of the ISKNV and LMBV genome did not change in over-expressing scTRIM44 cells. (**D**): The viral copies of the SCRV genome were markedly down-regulated in cells transfected with siTRIM44-3, but the viral copies of ISKNV and LMBV genome were no significant differences compared with NC group, NC: negative control. *: *p* < 0.05; ***: *p* < 0.001.

The copies of the SCRV genome were significantly up-regulated in over-expressing scTRIM44 cells; however, the copies of the SCRV genome were markedly decreased in knocked-down scTRIM44 cells by transfecting siRNA (Figure 6A,B). The copies of the ISKNV and LMBV genomes represented no significant change in over-expressed scTRIM44 cells (Figure 6A,B). After knocking down scTRIM44, there was also no significant change of the copies of the ISKNV and LMBV genomes (Figure 6A,B). These results indicated that scTRIM44 positively regulated SCRV infection but was not involved in ISKNV and LMBV infection.

### 3.6. scTRIM44 Positively Regulated the RIG-I- or MDA5-Mediated Interferon Pathway

**Figure 7:**
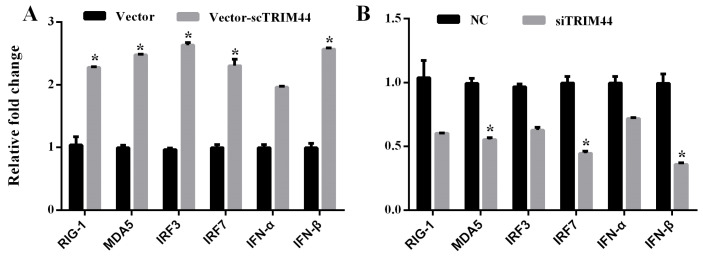
scTRIM44 positively regulated RIG-I- and MDA5-mediated interferon signaling molecules. (**A**): Compared to the cells transfected with the empty vector, the expression of RIG-I, MDA5, IRF3, IRF7, IFN-α, and IFN-β mRNA were up-regulated in over-expressiing scTRIM44 cells. (**B**): The expression of RIG-I, MDA5, IRF3, IRF7, IFN-α, and IFN-β mRNA expression were down-regulated in cells transfected with siTRIM44. NC: negative control. *: *p* < 0.5.

We detected the mRNA expression of RIG-I, MDA5, IRF3, IRF7, IFN-α, and IFN-β in over-expressing scTRIM44 or knocked-down scTRIM44 cells. The expression of RIG-I, IRF3, IRF7, IFN-α, and IFN-β in the mRNA level was significantly increased in over-expressed scTRIM44 cells (Figure 7). After knocking down scTRIM44, the expression of RIG-I, IRF3, IRF7, IFN-α, and IFN-β mRNA were significantly down-regulated (Figure 7). These results suggested that scTRIM44 may regulate the interferon pathway induced by RIG-I or MDA5.

## 4. Discussion

Many studies have indicated that TRIM family proteins possessed antiviral activity by directly effecting viral replication or via regulation of innate immune pathways [9,25]. Several fish TRIM proteins have proven to regulate fish viral infection through interferon-related signaling [26,27]. Here, the expression of scTRIM44 mRNA in many mandarin fish tissues can be detected, and it showed relatively high expression in immune-related organs. After ISKNV and LMBV infection, mRNA of scTRIM44 in spleen and cells appeared down-regulated, but the expression of scTRIM44 mRNA was up-regulated in CPB cells and spleen infected with SCRV. ScTRIM44 positively regulated SCRV infection but didn’t regulate ISKNV and SCRV infection. Meanwhile, scTRIM44 positively regulated the RIG-I- and MDA-5-mediated interferon pathways. These results suggested that scTRIM44 positively regulated SCRV infection through RIG-I- and MDA-5-mediated interferon signaling.

The C-terminal of the TRIM family protein consists of a RING zinc finger domain, one or two B-Box zinc finger structures, and a coiled-coil region, and the RING domain possesses E3 ubiquitin ligase activity [28]. TRIM44 is a member of the TRIM family, but it lacks the RING domain. The TRIM44 of orange spotted grouper contained the conserved B-Box domain and coiled-coil domain, but not a RING domain [21]. scTRIM44 also contained B-box-type zinc finger superfamily (Bbox_SF), one B-box domain (B, 166–207 aa), and the coiled-coil domain (CC, 279–309 aa), but lacked the canonical RING domain. The domains (B and CC) of scTRIM44 were 95.77%, 70.43, 56.34%, and 33.81% identical with *Micropterus dolomieu*, *Danio rerio*, *Rana temporaria*, and *Homo sapiens*, respectively. These results indicated that the variations in TRIM44 aa sequences were large among different species; however, the motif characteristics of fish TRIM44 structure was similar to that of mammal TRIM44, which indicated that scTRIM44 possessed a similar function to mammalian TRIM44.

The up-regulation of TRIM44 can be detected in various kinds of cancer, such as osteosarcoma, gastric cancer, lung cancer, breast cancer, papillary thyroid cancer, testicular germ cell tumor, gastric cancer, and prostate cancer, which suggested that TRIM44 presented a broad spectrum of expression in tissues [29,30]. ScTRIM44 mRNA can also be detected in different tissues, with especially high expression in immune organs; this phenomenon is similar to the expression of TRIM59 mRNA in mandarin fish [14]. The expression of scTRIM44 mRNA was significantly down-regulated in cells and spleen infected with ISKNV and LMBV, but the expression of scTRIM44 mRNA increased in cells and spleen post-SCRV infection. The changing trends of scTRIM44 mRNA was consistent with scTRIM59 infected with ISKNV [14]. These results indicated that scTRIM44 played different roles in ISKNV, LMBV, and SCRV infection.

Studies have suggested that TRIM44 played important regulatory roles in type I IFN signaling and antiviral immunity [16,31]. Yang et al. demonstrated that TRIM44 can positively regulate the virus-triggered immune response by enhancing the stability of VISA [19]. However, grouper EcTRIM44L was proven to increase virus replication by inhibiting the MDA5- or MAVS-induced interferon immune response [21]. The TRIM protein usually acts as ubiquitinase to regulating RIG-I and MDA5, which can recruite IRF3/IRF7 to promoting the production of type I interferons. In this study, scTRIM44 promoted the expression of RIG-I, MDA5, IRF3, IRF7, IFN-α, and IFN-β mRNA. This result indicated that scTRIM44 positively regulated RIG-I- or MDA5-mediated interferon immune response, which was consistent with Yang’s report. The expression of RGNNV CP and RdRp were significantly up-regulated in EcTRIM44L-over-expressing cells; however, the expression of SGIV MCP and VP19 showed no significant changes in EcTRIM44L-over-expressing cells [21]. In the present study, the copies of the SCRV genome were significantly increased in scTRIM44-over-expressing cells, but the copies of ISKNV and LMBV genome showed no significant alterations in over-expressing scTRIM44 cells. RGNNV and SCRV are both RNA viruses, while SGIV, ISKNV, and LMBV are double-stranded DNA viruses belonging to iridoviridae family [32]. These results showed that fish TRIM44 positively regulated fish RNA virus infection, but it showed no effect on fish double-stranded DNA viruses. The reason for this different antiviral activity of scTRIM44 against fish RNA viruses and double-stranded DNA viruses will need further studies.

Overall, scTRIM44 aa was 90.90%, 42.89%, 26%, and 23% identical with *Micropterus dolomieu*, *Danio rerio*, *Rana temporaria*, and *Homo sapiens*, respectively. The scTRIM44 consisted of one B-box domain, and a coiled-coil domain, but not the RING domain. The expression of scTRIM44 mRNA was significantly down-regulated in cells and spleen infected with ISKNV and LMBV, but the expression of scTRIM44 mRNA increased in cells and spleen post-SCRV infection. scTRIM44 negatively regulated RIG-I- or MDA5-mediated interferon immune response. The viral copies of the SCRV genome were significantly increased in scTRIM44-over-expressing cells, but there was no significant change of the expression of the ISKNV and LMBV genome in over-expressing scTRIM44 cells. These results suggested that fish TRIM44 positively regulated fish RNA virus infection, but there was no influence on fish double-stranded DNA virus infection. This data provided the basis for further studying the antiviral mechanism of scTRIM44 and a novel insight into the antiviral activity of TRIM44 through the interferon response signaling.

## Figures and Tables

**Table 1:** The primers used in this study.

Primer Name	Sequence	
TRIM44-F	5′ ATGGACCACAAAGGGGAAC 3′	
TRIM44-R	5′ TCAGGGGGCGTGGTCCATG 3′	
pCMV-TRIM44-F	5′ CG**GAATTC**ATCGACCACAAAGGGGAAC 3′	EcoRI
pCMV-TRIM44-R	5′ GG**GGTACC**TCAGGGGGCGTGGTCCATC 3′	KpnI
ScTRIM44-RT-F	ACTTGGCACCAAAAGAGACTCC	
ScTRIM44-RT-R	TCTCACTGTGTCCCTCTTCCCA	
TRIM44-1-sense	GCUGAAACAAGAGGAACUUTT	Trim44-siRNA
TRIM44-1-antisense	UAGUGCCAAGUCCAUUAGCTT	
TRIM44-2-sense	GCUAAUGGACUUGGCACUATT	
TRIM44-2-antisense	UAGUGCCAAGUCCAUUAGCTT	
TRIM44-3-sense	GGAGGAGAAGAGGACCCUUTT	
TRIM44-3-antisense	AAGGGUCCUCUUCUCCUCCTT	
NC-sense	UUCUCCGAACGUGUCACGUTT	
NC-antisense	ACGUGACACGUUCGGAGAATT	
IRF3-RT-F	GTCTACAGCCCTGAACTCAACGG	RT-PCR
IRF3-RT-R	AAATCTCTTGGGGCTGTGTGGTC	
IRF7-RT-F	AGTTCACCTCTGCAGCCATGTAT	
IRF7-RT-R	GTTAAGGACGCGGTTGGTGAAAT	
RIG-I-RT-F	AAGTGCAAGATGTTTGCGTGTC	
RIG-I-RT-R	GAAGTTGATGGGCTTTCTGTGAG	
MAD5-RT-F	CTCCCGACAGGAAGTGGTAAA	
MAD5-RT-R	GCGGAATAATGCTGCTCAAC	
IFN-α-RT-F	TGAGGATGCTGGAGTGACC	
IFN-α-RT-R	GCCTGCCGAGTAACATTGAC	
IFN-β-RT-F	ACGGATCTCAAGTCAGGGTC	
IFN-β-RT-R	TGAGTAGGGTATGAGGGCATT	
18S-F	CATTCGTATTGTGCCGCTAGA	
18S-R	CAAATGCTTTCGCTTTGGTC	

## Data Availability

The datasets used and/or analyzed during the current study are available from the corresponding author upon reasonable request.
